# Extraction of Mycosporine-like Amino Acids and Proteins from the Agarophyte *Gelidium corneum* Using Pulsed Power Techniques

**DOI:** 10.3390/foods12071473

**Published:** 2023-03-30

**Authors:** Colin McReynolds, Amandine Adrien, Antoine Silvestre de Ferron, Nadia Boussetta, Nabil Grimi, Laurent Pecastaing, Susana C. M. Fernandes

**Affiliations:** 1 IPREM—Institute of Analytical Sciences and Physico-Chemistry for Environment and Materials, E2S UPPA: Energy Environment Solutions, Université de Pau et des Pays de l’Adour, CNRS—Centre National de la Recherche Scientifique, 64600 Anglet, France; 2 MANTA—Marine Materials Research Group, Universite de Pau et des Pays de l’Adour, E2S UPPA, 64600 Anglet, France; 3 Laboratoire des Sciences de l’Ingénieur Appliquées à la Mécanique et au Génie Électrique—Fédération IPRA, Université de Pau et des Pays de l’Adour/E2S UPPA, EA4581, 64000 Pau, France; 4 TIMR (Integrated Transformations of Renewable Matter), ESCOM, Université de Technologie de Compiègne, Sorbonne Universités, Centre de Recherche Royallieu, CEDEX CS 60319, 60203 Compiègne, France

**Keywords:** red algae, seaweeds, mycosporine-like amino acids, proteins, marine bioresources, valorization, sustainable extractions, high-voltage electrical discharges, pulsed electrical fields

## Abstract

*Gelidium corneum* (syn. *sesquipedale*) is an industrially and ecologically important species of red alga used for the production of high-quality agar. However, the species is also of growing interest for the production of other valuable compounds, such as mycosporine-like amino acids (MAAs), with potential cosmeceutical and biomedical applications. Novel methods using two pulsed power techniques, high-voltage electrical discharges (HVED) and pulsed electrical fields (PEF), were evaluated for efficacy of MAA extraction. Algal suspensions were prepared at two ratios (1:20 and 1:40 *w*:*v*). Four different extraction protocols were compared: (i) high-voltage electrical discharges, (ii) pulsed electric fields, (iii) maceration at room temperature, and (iv) maceration at 50 °C. The algae were treated in three states: freshly harvested, dried, and powdered. HVED and PEF treatments were effective when performed on fresh algae, and in particular the HVED treatment resulted in yields of MAAs twenty times higher than the control: 0.81 ± 0.05 mg/g_Dry Weight (DW)_ vs. 0.037 ± 0.002 mg/g_DW_. This effect was not observed to the same extent when the algae were dried or powdered, although HVED remained the most selective method overall.

## 1. Introduction

The *Gelidium corneum* (syn. *sesquipedale*) (Hudson) J.V.Lamouroux 1813 is a species of red algae occurring along the coasts of northeastern Africa to the British Isles. *Gelidium corneum* (GC) and related Gelidiales are valuable seaweeds and have been the subject of commercial exploitation around the world for many decades for their high-quality agar and agarose for applications in food, cosmetics, and biotechnology [1,2,3,4]. Most of the global production of GC takes place in northern Spain and Morocco [5], although it is also harvested in southwest France [6] and Portugal [7]. Commercial pressure and climate change pose a threat to the species, which is a key part of coastal ecosystems [8,9], leading to harvest restrictions and supply shortages with global implications [10]. Improved use of the non-agar fraction of the biomass may sustain continued, potentially limited, exploitation of the species and provide new opportunities for bio-based materials [11].

Indeed, GC has been shown to be a promising source of a variety of compounds beyond agar for the production of bio-based materials; several alternative commercial products produced using GC already exist, including multiple patented extracts with antioxidant [12], wound healing [13], and UV-blocking [14] properties. Recent interest in the species has focused on the residues of agar/agarose production as a source of valuable compounds [11], from precursor oligosaccharides [15] to pyrolyzed char [16]. However, GC also contains potentially valuable proteins [7], cellulose [17], and smaller molecules such as mycosporine-like amino acids (MAAs) [6]. 

In GC, the MAAs asterina 330, palythine, shinorine, and porphyra-334 have all been detected, with asterina being the dominant MAA [6,18,19]. Potential applications include integration into next-generation biomimetic UV-protective materials [20] to mitigate problems caused by synthetic sunscreens in coastal areas [21,22,23], for example.

For efficient extraction of these molecules, cellular membranes and tissue structure must be disrupted to enable their diffusion into the extraction solvent. It requires low temperatures and suitable solvents: MAAs are possibly entirely denatured by the prolonged high temperatures and high pH used in agar production [3]. As such, there is a need to develop extraction methods compatible with MAAs and the other compounds of commercial interest in GC. Emerging methods for treating algae are high-voltage electrical discharges (HVED) and pulsed electrical fields (PEF), environmentally friendly methods involving low total energy input and which do not require high temperatures or harsh solvents. Both methods use high-power electrical pulses to facilitate extraction, although the mechanisms differ. HVED involves liquid phase discharges and associated physical phenomena—pressure shock waves, flashes of UV light, and electrochemical reactions [24,25,26]—to disassociate biological membranes and release intercellular materials. PEF treatment consists of the application of electrical pulses of short duration (nanosecond to millisecond scale) and varying in strength from 100 V/cm to 80 kV/cm [27]. The procedure is also known by the name of its principal mechanism of extraction, electroporation [28], in which permeability of the cell membrane is increased [29]. Studies on the use of these physical pretreatment methods on macroalgae are still scarce, with most research focusing on electroporation. Indeed, work on the green algae *Ulva* sp. showed improved protein yield [30], improved carbohydrate yield [31,32], improved functionality (antioxidant activity) [33], and efficient ash removal [34] when using PEF in the extraction process. Other authors working on green, red, and brown algae showed fast extraction times and identical performance of PEF compared to hot water extracts [35]. To the best of our knowledge, at the time of writing no studies investigating the potential of HVED treatment for macroalgal compounds have yet been published.

Herein, the possibility of extracting MAAs from untreated GC using HVED and PEF was investigated. Here, energy input, in terms of kJ/kg solution (kg_ool_) was used as a basis of comparison to evaluate the efficacy of the two methods, and compared to unheated and heated controls. GC is typically harvested and transported, then air-dried in fields or covered hangars before further processing. Raw GC is sold to producers in both dry and fresh forms, although dry is preferred, when possible, as a large portion of fresh biomass is composed of water thus increasing weight, volume and, by extension, transport costs. For active molecules, the extraction of algal metabolites usually starts with freeze-drying or grinding of the algae in some manner [36] but this is not the case with algal biopolymers such as agar, where algae are minimally pre-processed to reduce costs [3,5]. To investigate the differences incurred by the state of the algae for the eventual application of HVED and PEF, the effect of these different parameters (fresh, dry, and powdered) was considered. In addition to MAAs, the protein fraction of the extract was also examined, as the methods have previously been shown to be suitable for the extraction of this type of molecule, and red algae proteins show interesting amino acid profiles for inclusion in functional foods and feed [37]. The antioxidant capacity of each extract was also examined, to explore the functionality of raw extracts for direct use in foods, cosmetics, or other applications. With a suitable method, these molecules may be extracted alongside the other high-value products in the algae as part of a biorefinery approach, enabling the reducing of harvesting pressure on the algae while maintaining its economic importance.

## 2. Material and Methods

### 2.1. Collection and Preparation of Algal Material 

Algal material was collected between 2020 and 2021 from local fishermen at the port of Ciboure St-Jean de Luz, France, and transported to the laboratory. Fresh seaweed was briefly rinsed (<1 min) with running tap water to remove visible sand particles and epiphytes, and centrifuged using a salad spinner for 1 min to remove surface water. For experiments on dry algae, the thalli were then set to air-dry on racks in a well-ventilated, dry room at 25 °C until constant weight. Room-temperature air-dried GC (hereafter referred to as “dry” samples) was then transferred to a resealable plastic bag and stored in a cool, dark container until experimentation. Prior to HVED and PEF treatment, fresh and dry GC thalli were chopped into approximately 2.5 cm pieces. Powdered GC (hereafter referred to as “powder” samples) was obtained by freeze-drying dry seaweeds with a CRIOS −55 °C countertop freeze-drier (Cryotec, Saint Gély du Fesc, France) and ball-milling for 9 min in a RETSCH PM100 ball mill (Haan, Germany), alternating the direction of rotation in 3-min cycles. Powdered seaweeds were conserved in airtight containers in the dark at 4 °C until use. To summarize, 3 different forms of raw GC were used: fresh, dry, and powder.

### 2.2. Characterization of the Raw Material

The moisture content of each form of GC was determined by oven-drying the samples at 105 °C overnight. The nitrogen content of the initial biomass was determined using a FLASHEA 1112 elemental analyzer (Delft, The Netherlands). The equipment was calibrated using BBOT (2, 5-bis (5-tert-butyl-benzoxazol-2-yl)-thiopen, C_26_H_26_N_2_O_2_S, Thermo Fisher, Illkirch-Graffenstaden, France) and sulphanilamide (4-aminobenzenesulfonamide, C_6_H_8_N_2_O_2_S, Thermo Fisher, Illkirch-Graffenstaden, France) standards in linear calibration mode. Samples weighing 1.5 mg were mixed with 5 mg vanadium pentoxide and incinerated at 900 °C on a He carrier gas. Eager 300 software was used to obtain N content. Total protein content of the raw material (16.71 ± 0.37%) was estimated by multiplying the N content by 5, the conversion factor for this type of macroalgae (Rhodophyta) as determined by Angel et al. [38]. Fresh GC presented a moisture content of 71.37 ± 0.58% (n = 3), dry seaweed 13.33 ± 2.32% (n = 3), and powdered 4.96 ± 0.16% (n = 3).

### 2.3. Extraction Protocols

Algal suspensions were prepared at 1:20 and 1:40 (*w*_dry_/*v*) solid to liquid ratios with distilled water prior to insertion in PEF and HVED. To compare algae in different states (fresh, dry, and powder), an equivalent dry weight was used for each treatment with the moisture content of the algae considered part of the liquid ratio. Furthermore, room-temperature dried algae were allowed to rehydrate in the reaction medium for 30 min with magnetic agitation (400 RPM) before the treatment to restore turgescence.

#### 2.3.1. PEF- and HVED-Assisted Extraction

PEF treatments were applied using a PEF generator (Basis, Saint-Quentin, France) and a 1 L cylindrical treatment chamber with two plane stainless electrodes (12.6 mm diameter). The generator provided monopolar, exponential pulses. The voltage and current delivered by the high-power supply were, respectively, 40 kV and 10 kA. The distance between electrodes was 2 cm. The corresponding field intensity was 20 kV/cm. The pulse duration was 5 µs. The pulse energy was 200 J. The pulse frequency was 1 Hz. A high voltage probe (ROSS VD45, 2 MHz) and a current probe (Pearson 3972, 20 MHz) were linked to a Tektronic TDS1002 (Beaverton, USA) oscilloscope in order to monitor voltage and current profiles during the treatment.

The total algal suspension weight was 0.25 kg. Algal suspensions were treated with up to 625 pulses which corresponded to a total specific energy input of *E_tot_* = 500 kJ/kg_sol_, according to Equations (1) and (2) below. Samples (0.5 mL) were taken during treatment at 0, 50, 100, 250, 350, and 500 kJ/kg_sol_ to track MAA diffusion. After treatment, the algal suspension was transferred to a beaker to macerate at room temperature for a further 2 h under magnetic agitation (400 RPM) with 0.5 mL samples taken every 30 min. All treatment conditions were performed in duplicate.

(1) $$E_{tot}=\sum E/M_{sol}$$

with:

(2) $$E=0.5\times C\times V^{2}$$

where *E* = energy (J) per pulse and *M_sol_* = Mass of suspension (algae + distilled water), *C* = Capacitance of the generator (F) and *V* = Applied voltage (Volts).

The HVED device was designed by the SIAME laboratory (Pau, France), using a 50 kV/100 mA power supply operating at 40 kV/ 30 mA to produce pulses at 1 Hz, with a 1.2 µF capacitor and home-made triggering spark gap. The electrodes used were in point–point configuration with an inter-electrode distance of 8 mm. The pulse energy was 750 J (mean value). Algal suspensions were treated with up to 300 pulses which corresponded to a specific energy input of 500 kJ/kg_sol_. The total suspension mass was 0.45 kg. As described above for the PEF treatment and at the same intervals, 0.5 mL samples were taken during HVED treatment to track molecular diffusion from the seaweed to the solution during both the application of pulses and during the maceration that followed. The amplitude of the pressure waves generated during HVED was calculated according to the Equation (3) proposed by Bacqueyrisses et al. [39].

(3) $$p_{peak}=k\times \frac{I_{max}}{r}\times \sqrt{\frac{d}{{\left(t_{char}\right)}^{0.68}}}$$

where *p_peak_* is the peak pressure (Mpa, bar), *k* is a constant dependent on the characteristics of the fluid (here it will take the value 28 as determined experimentally [39]), *I_max_* is the peak current (A), *r* the distance from the discharge (m), *d* the inter-electrode gap spacing (m), and t_char_ the time (s) between the discharge and peak current with the factor 0.68 determined experimentally. After HVED pretreatment, the algal suspensions were transferred to beakers to macerate at room temperature for a further 2 h under magnetic agitation (400 RPM). All treatment conditions were performed at least in duplicate.

#### 2.3.2. Control Extraction

Control extractions without electrical pulses applied were performed to isolate the effects of the HVED and PEF against the effects of simple maceration with agitation and thermal effects. Macerations at room temperature (MRT) were performed with fresh, dry, and powder samples. Algal material was introduced in equivalent volumes as described above: 1:20 and 1:40 (*w*/*v*) ratios for equivalent periods of extraction under magnetic stirring at room temperature. Thirty minutes of maceration with magnetic agitation (400 RPM) was added to account for diffusion during the other types of treatment.

Taking the Joule effect and specific enthalpy of water into account, an increase in the suspension temperature, taking it to 50 °C, was expected after PEF pretreatment and, as such, a heated control was performed. Maceration at 50 °C (M50) involved heating the algal suspension to 50 °C over 30 min on a magnetic stirring plate. Then the algal suspension was allowed to macerate at room temperature for a further 2 h under magnetic agitation (400 RPM).

A summary of the different extraction conditions is provided in Table 1. After extraction, all algal suspensions were filtered through fine nylon mesh and conserved at −25 °C until further analysis.

### 2.4. Characterization of the Extract Solutions

#### 2.4.1. Monitoring of the Extraction

To evaluate the presence of MAAs and proteins during the treatment and maceration, 100 µL samples were collected from the extraction media and spectra were measured from 250 to 750 nm using a dual-beam ultraviolet–visible spectrophotometer (Thermo Scientific™ Multiskan Sky, Thermo Fisher, Illkirch-Graffenstaden, France). To evaluate the MAA diffusion, a full spectrum absorbance was recorded and 325 nm was selected as the wavelength indicative of the presence of MAAs in the extract.

#### 2.4.2. Dry Weight of the Extracts

For the determination of the extract dry weight material *m_E/DW_* (mg/g_DW_), frozen extraction solutions were allowed to thaw for 24 h. The solutions were shaken for 1 min and then centrifuged for 1 min to separate insoluble matter. An amount of 10 mL of the supernatant was then freeze-dried for at least 72 h with a CRIOS −55 °C countertop freeze-drier (Cyrotec, Saint Gély du Fesc, France). Total soluble material was determined using the following Equation (4):

(4) $$m_{E/DW}=\frac{m_{se}\times \left(\frac{V_{E}}{V_{S}}\right)}{m_{DW}}$$

where *m_se_* expresses the soluble material in the subsample (mg), *V_E_* the total volume of extract (mL), *V_S_* the volume of the freeze-dried subsample (10 mL), and *m_DW_* the dry weight of the GC used in the extraction. Measurements were performed in triplicate.

#### 2.4.3. MAA Profiling and Quantification

MAA profiling and quantification of algal extracts were based on the untargeted hydrophilic interaction liquid chromatography using multistage electrospray mass spectrometry Electrospray Orbitrap MS^2^/MS^3^ analysis developed by Parailloux et al. [19]. In summary, samples were diluted 100-fold in [80:20] acetonitrile:5 mM ammonium acetate pH = 5.3 (*v*/*v*), and centrifuged for 10 min at 12,000 rpm. A 10 µL aliquot was then injected into the LC–MS system. Separation was carried out on an Ultimate 3000 RSLC system (Thermo Fisher Scientific) with a CAPCELL CORE PC column (OSAKA SODA) and the following mobile phases: A: acetonitrile; B: 5 mM ammonium acetate pH = 5.3. Detection was ensured by an Orbitrap Fusion Lumos (Thermo Fisher Scientific) high-resolution mass spectrometer. The acquisition consisted of a full MS at R = 120,000 and ddMS2 scans. Quantification was achieved by external calibration based on the full MS signal using 5 mg/L shinorine and palythine standard solutions. The quantification of asterina-330 was estimated assuming an electrospray MS response factor similar (within 10%) to the standards [6].

#### 2.4.4. Protein Quantification

Protein content of the extracts was quantified using a modified version of the Lowry method adapted for use with 96-well microplates [40]. Bovine serum albumin was used as standard (0–0.5 mg/mL). In summary, 20 μL of blank, samples or standard were added to the plate followed by 200 μL of Lowry’s solution. The plate was incubated for 20 min at room temperature in the dark and 20 μL of Folin reagent was then added. The plate was incubated for a further 35 min at room temperature and the absorbance was read at 750 nm. Extract protein yield *(%Protein*) Equation (5) was obtained by dividing the weight of extracted protein (*m_protein_*) by total dry weight of extracts (*m_DW_*) and the yield of extractible protein *(%Extracted protein*—Equation (6) was obtained by dividing extract protein content by total protein of algae (*N_protein_*) obtained by elemental analysis (see Section 2.2 above)

(5) $$\%Protein=\frac{m_{protein}}{m_{DW}}$$

(6) $$\%Extracted\;protein=\frac{\%Protein}{N_{protein}}$$

#### 2.4.5. FRAP Antioxidant Capacity

The antioxidant activity of the seaweed extracts was measured using a ferric reducing antioxidant power (FRAP) assay according to the Benzie and Strain method, with slight modification for use with a 96-well plate [6,41,42]. A working FRAP solution was first prepared by mixing 300 mM acetate buffer (pH 3.6), 10 mM TPTZ, 40 mM HCl, and 20 mM FeCl_3_ at a ratio of 10:1:1 (*v*/*v*/*v*) and warming it to 37 °C for 10 min. Next, 20 µL of sample solution was added to the wells, and the reaction was started by adding 200 µL of the warm FRAP solution. The reaction mixture was incubated in darkness at room temperature for 10 min and the absorbance was measured at 593 nm. Trolox standard was used to make a standard curve and results were expressed as µg Trolox equivalents (TE) per g of dry algae (µg TE/g _DW_).

#### 2.4.6. Statistical Analysis

Type III ANOVA with Tukey post-hoc test statistical analysis was performed using JASP (Version 0.17.1) (Amsterdam, The Netherlands).

## 3. Results and Discussion

### 3.1. Thermal and Physical Effects of PEF and HVED

Due to the high intensity of the treatment, PEF pretreatment resulted in heating as a result of the transfer of energy to the aqueous medium. As such, temperature increases of up to 43 °C were observed over the course of treatment (Supplementary Information Table S1).

**Figure 1:**
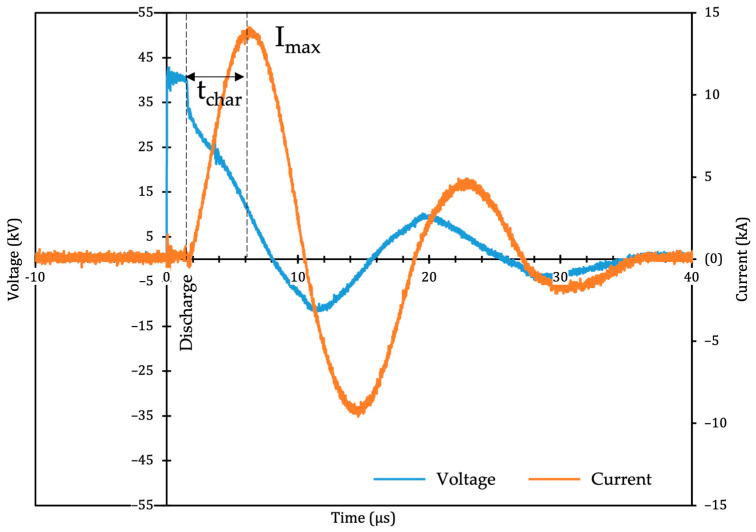
Typical voltage and current recordings for a single electrical discharge. *t_char_*: time (s) between the discharge and peak current *I_max_*.

During HVED pretreatment, average peak current *I_max_* was 12.29 ± 1.40 kA, with *t_char_* circa 3.9 µs (see sample oscillogram in Figure 1). The experimental arrangement was very similar to that presented by Bacqueyrisses et al. in [39]; the constant values (k and 0.68 in Equation (3)) previously determined with hydrophone in [39] can be taken for the calculation of the peak pressure wave. Given the experimental setup, the amplitude of the peak pressure wave generated during an average discharge was calculated to be circa 13.61 Mpa at a distance of 45 mm (corresponding to the radius of the treatment cylinder). It has been proven in [43] that the geometry of the treatment chamber enabling reflections of shockwaves can play a role in the extraction process with higher energy efficiency for polyphenol yields from grape pomace in scaling-up trials. This assumption should likewise be taken into consideration due to the amplitude of the values reached here.

For HVED pretreatments, the increase in temperature was not as marked as that observed in PEF pretreatment. Although the electrical discharges result in highly localized extreme temperatures, the thermal inertia and larger volume of the treatment chamber allowed for effective dissipation of the heat. Heating was observed, however, with lower temperature increases than PEF: up to 16 °C for maximum attained temperatures of 35 °C (Supplementary Information Table S1). The duration of treatment varied slightly between experiments, due to the configuration of each device. Strong erosion of the electrodes was also noted during HVED treatment (approximately 0.6 mg/discharge, determined gravimetrically before and after a typical treatment run). This phenomenon may be reduced through the use of alternative materials such as tungsten or molybdenum [44].

The techniques applied here may be further optimized for specific energy input. Total energy input per kilogram of raw material reached 25 kJ/kg algae dry weight (for a 1:20 ratio). Compared to other treatment methods, this is at the low end of the scale for mechanical treatment (20–40 kJ/kg), heating, or freezing/thawing (>100 kJ/kg) for fruit/vegetable tissues [45].

### 3.2. Effect of PEF and HVED on Total Soluble Compounds

The rate of the extraction processes, with or without (control) electric pulse treatment, was monitored by measuring the UV absorbance at 325 nm (characteristic wavelength of MAAs) of the solution throughout the steps of the processes (Supplementary Information Figure S1). The results showed a fast release of the absorbing compounds at 325 nm for all forms of GC and for every treatment, except for those undertaken at room temperature with fresh algae. Indeed, at least 80% of the maximum absorbance values were reached within the first 30 min of extraction. These results highlight the possible effect of initial washing: in the agar industry, GC is frequently handled dry with solid contaminants present (sand) [46,47]. These contaminants are removed through washing with water, which may result in the concomitant elution of a significant portion of MAAs.

Post-extraction, the different samples were visibly different according to the treatment type and state of the algae (Supplementary Information Figure S2). Powder algae extracts showed a noticeable darker color (red-orange) compared to the dry or fresh GC, indicating possible co-extraction of colored compounds such as phycobiliproteins. This visual observation was confirmed by the analysis of the full UV spectra (Supplementary Information Figure S3) that showed other notable peaks besides the peak at 325 nm. The spectra show a large peak in the short UVB region due to the presence of proteins and DNA as well as several small peaks in the 500–600 nm region, indicating phycobiliprotein peptides [48], which are notably present in the extracts with a visible reddish hue. 

**Figure 2:**
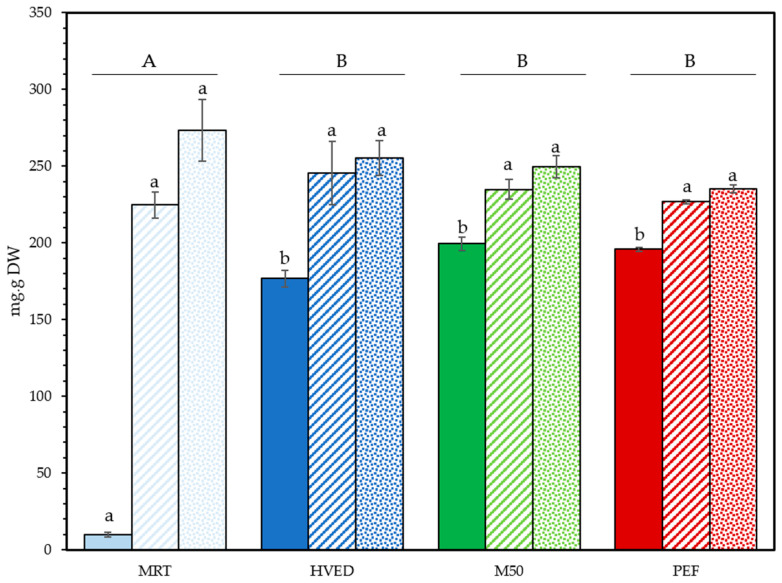
Final dry weight of soluble compounds extracted from GC. Complete fill: fresh GS; dashed fill: dry GS; dotted fill: powder GS. Data are shown as mean ± SD (n = 3). Upper case letters display differences between treatments (values averaged across state, n = 12). Lower case letters denote significant differences between treatments by state n = 3). Type III ANOVA with Tukey post-hoc test.

Final yields of soluble dry matter in algal extracts varied according to the initial state of the algae (Figure 2). Yields varied the most in fresh GS, yet the control group was the only one significantly different from the rest with just 9.72 ± 1.53 mg/g_DW_ extract dry weight (type III ANOVA with Tukey post-hoc test). No differences were observed between treatment types for dry and powder GS, suggesting that diffusion of water-soluble compounds was enabled by processes affecting the cell wall during desiccation. Powdered GS showed the highest overall average yields of 257.37 ± 19.76 mg/g_Dw_, the small particle size enabling high contact area for the transfer of soluble compounds to the solution. Yields represented close to 27% of the initial dry weight of GS. Concerning the ratio, 1:40 *w*/*v* did not yield significantly different means (Kruskal–Wallis test *p* = 0.875) and the same patterns were observed (Supplementary Information Figure S4).

### 3.3. MAA Identification and Quantification: Influence of the Electrical Treatments on the MAA Extraction Yield

Extraction efficacy was determined quantitatively by ddMS^2^/MS^3^ analysis from 1:20 extracts post-treatment and maceration, focusing on the most predominant MAAs: asterina-330, palythine, and shinorine [6,19]. Annotation of candidate compounds was based on a set of fragment ions, and neutral and radical losses specific to their fragmentation pathways acquired in positive ionization mode. Although other MAAs, namely porphyra 334, were detected, generally they represented less than 0.5% of total MAAs in GC and were not quantified here [6].

**Figure 3:**
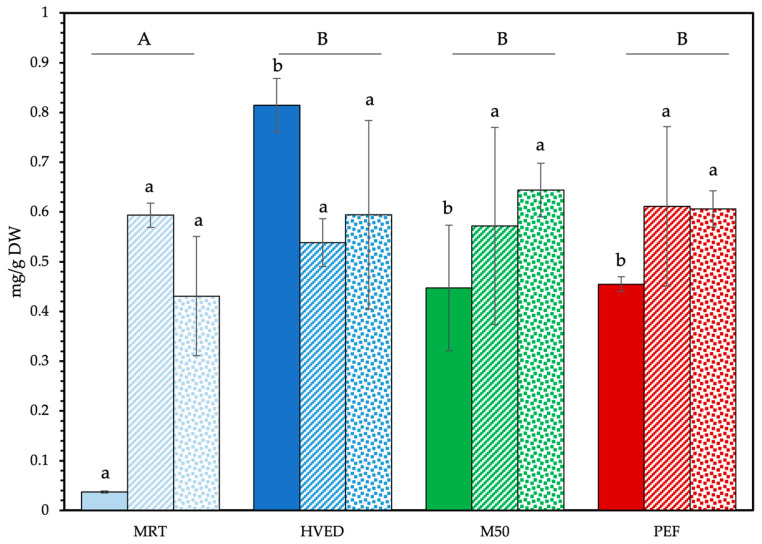
Final total MAA yields. Solid fill: fresh GC; dashed fill: dry GC; dotted fill: powder GC. Upper case letters display differences between treatments (values averaged across state, n = 12). Lower case letters denote significant differences between treatments by sate n = 3). Type III ANOVA with Tukey post-hoc test.

Final MAA content varied according to the state of the algae and the applied pretreatment (Figure 3 and Table 2). The most prominent differences in extraction efficacy were observed in fresh GC. The control maceration at room temperature yielded 0.04 ± 0.00 mg/g_DW_ of total MAAs, whereas the HVED-treated sample yielded 0.81 ± 0.05 mg/g_DW_, a twentyfold increase. Maceration at 50 °C (M50) and PEF treatment resulted in nearly identical yields of 0.45 ± 0.13 and 0.45 ± 0.01 mg/g_DW_, respectively: a twelvefold increase versus the control. For dry and powder GC, the differences between treatment types were less marked. With dry GC, the controls had 0.59 ± 0.02 mg/g_DW_, and means in pretreated samples varied from 0.54 ± 0.05 mg/g_DW_ in HVED-pretreated samples to 0.61 ± 0.16 mg/g_DW_ total MAAs in PEF samples. As such, the pretreatment did not result in higher overall yields. Similar observations could be made for powder GC, although MAA yields from pretreated samples were greater than those of the control. The maceration at room temperature (MRT) had the lowest overall mean (0.43 ± 0.12 mg/g_DW_), whereas the HVED pretreatment resulted in MAA yields of 0.59 ± 0.19 mg/g_DW_, M50 in yields of 0.64 ± 0.05 mg/g_DW_ and PEF in yields of 0.61 ± 0.04 mg/g_DW_. As such, the elevation of temperature showed a similar effect to HVED and PEF on the diffusion of the MAAs from the powder.

The most notable differences were observed between fresh and dried algae. In fresh algae, the physical phenomena induced by HVED (shock waves) induced the most damage to algal thalli and consequent MAA release. For the other samples (M50 and PEF), elevated temperatures and pulsed electrical fields are known to weaken the structure of cell walls, resulting in softer and less rigid texture, and enhanced permeability [29,49]. They also increase the free amino acid content in red algae extracts [50]. No significant differences were observed between treatments on dried algae, either whole or as freeze-dried powder. The cell damage induced by the initial slow desiccation of these samples may have been sufficient to allow for equivalent diffusion of MAAs into the solution despite different treatment conditions. Indeed, desiccation has previously been shown to enhance membrane permeability in intertidal red algae, notably increasing amino acid leakage [51,52,53]. The similar results obtained for dry GC and powder GC also highlighted that granulometry obtained through the ball-milling protocol had little impact on MAA extraction yields.

These results were consistent with those obtained by Castejon et al. [6], who studied the effects of ultrasound-assisted extraction from the same algae in freeze-dried and powdered form. They obtained yields up to 1.38 mg/g_DW_ total MAAs when using longer maceration times (5 h vs. 2 h used here), with ultrasound-assisted extraction yielding up to 1.27 mg/g_DW_ depending on the temperature. Notably, short extractions starting at room temperature yielded the highest concentrations, and those starting at 40 °C yielded the lowest. This reflected the results presented here, as ultrasound-assisted extraction functions in a similar manner to HVED: high-intensity vibrations create cavitation and extreme pressure gradients, causing physical damage to algal cells [54]. Quintano et al. [55] recovered lower yields with methanolic extraction solvents in a similar biotope/latitude in northern Spain, although the specimens in that study were gathered manually at depth, which can negatively affect MAA concentrations [56,57].

The relative content of MAAs in the dry extract was then extrapolated from the concentration measured by MS/MS² (Table 3). HVED displayed the highest selectivity among all treated samples. This was especially apparent in fresh GC, where relative MAA content constituted nearly 11% of the freeze-dried extract compared to less than 5% for every other treatment and alga form.

### 3.4. Protein Quantification

**Figure 4:**
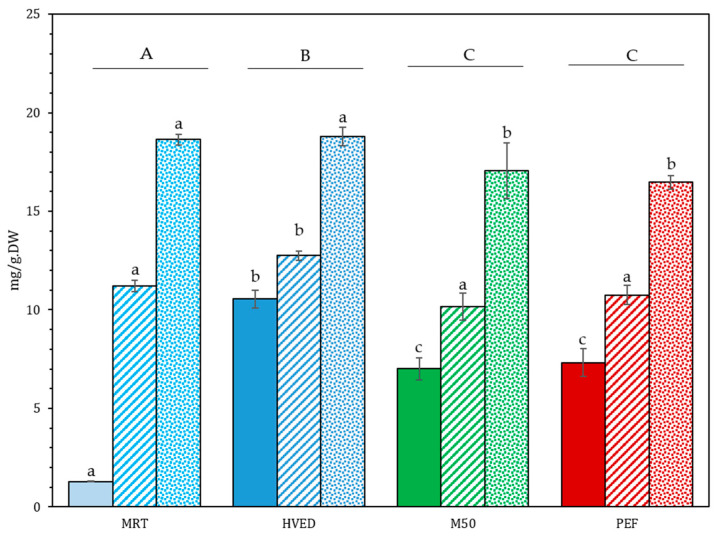
Final extracted protein yields in different treatments. Ratio dry algae/solvent of 1:20 (*w*/*v*). Solid fill: fresh GC; dashed fill: dry GC; dotted fill: powdered GC. Data are shown as mean ± SD (n = 4). Upper case letters signify differences between treatments (values averaged across state). Lower case letters denote significant differences between treatments by state. Type III ANOVA with Tukey post-hoc test.

The final extracted protein yields are presented in Figure 4. The relative proportion of proteins in the dry extract and of total extracted proteins are presented in Table 4. Total protein on the raw biomass was found to be 16.71 ± 0.37% (dw), corresponding well with the value of 16.25 ± 0.33% found by Cavaco et al., in Portuguese GC specimens [7].

Overall, the protein extraction yields showed a similar response to that of the MAAs, varying according to the algal state and the treatment type (Figure 4). The lower ratio of algae to solvent (1:40) resulted in universally higher yields (Supplementary Information Figure S5 and Table S2), possibly due to solvent saturation in the 1:20 ratio. Whatever the extraction conditions, powdered GC showed the highest extraction yields with values between 16.47 ± 0.33 and 18.78 ± 0.46 mg/g_DW_, showing that the granulometry was the main factor influencing the protein extraction yield. Dry GC also showed higher extraction yields than the fresh GC for every treatment, suggesting that the damage incurred on the cell wall due to desiccation-related processes had a significant impact on the protein extraction.

For the powder and dry GC, protein extraction yields were little affected by the treatment type. Indeed, for the dry GC, only the algae treated with HVED showed a significantly higher yield than the other treatments with an extraction yield of 12.63 ± 0.23 mg/g_DW_, (a 15% increase compared to the other treatments). For the powder GC, treatments that incurred an increase in temperature to 50 °C and above (M50 and PEF) showed a slight decrease in the extraction yield, of around 10%, compared to the MRT and HVED treatments. These temperatures may have adversely affected protein yield by denaturing temperature-sensitive proteins, changing conformation and reducing solubility [58].

For the fresh GC, the protein yields of MRT were significantly lower than all others, with only 1.29 mg/g_DW_. HVED pretreatment yielded the highest quantity of extracted proteins with 10.55 mg/g_DW_, significantly higher than the other treatments, and more than eight times higher than the control yield (ANOVA type III, Tukey post-hoc). In fresh M50 and PEF extracts, the protein extraction yield was lower and equivalent in value for both pretreatments: 7.01 and 7.32 mg/g_DW_, respectively.

In terms of relative content in the freeze-dried extract (Table 4), proteins represented only a small fraction of the total weight, generally under 10%. The highest relative content was observed with maceration at room temperature (13.3 ± 0.2%). However, this sample also had a very small total dry weight and the lowest overall protein content (1.29 + 0.01 mg/g_DW_) (Figure 4). HVED displayed the highest relative protein content across of all the treated samples and for all states of the GC. This is most likely due to the cell fragmentation induced by the shockwaves as proteins make up a considerable portion of the algal cell wall [37,59,60,61]. The proportion of total protein extracted via these methods was directly proportional to protein content in mg/g_DW_: between 1% (MRT, fresh GC) and 11% of total protein (MRT, HVED, powdered) were extracted from biomass.

Algal proteins have myriad uses, from foods for humans and animals [37,62], to bioactive peptides with medical applications [63]. As such, if the treatment method effectively extracts proteins, it may be of interest for completely valorizing GC biomass [64].

### 3.5. Antioxidant Activity of the Extracts

**Figure 5:**
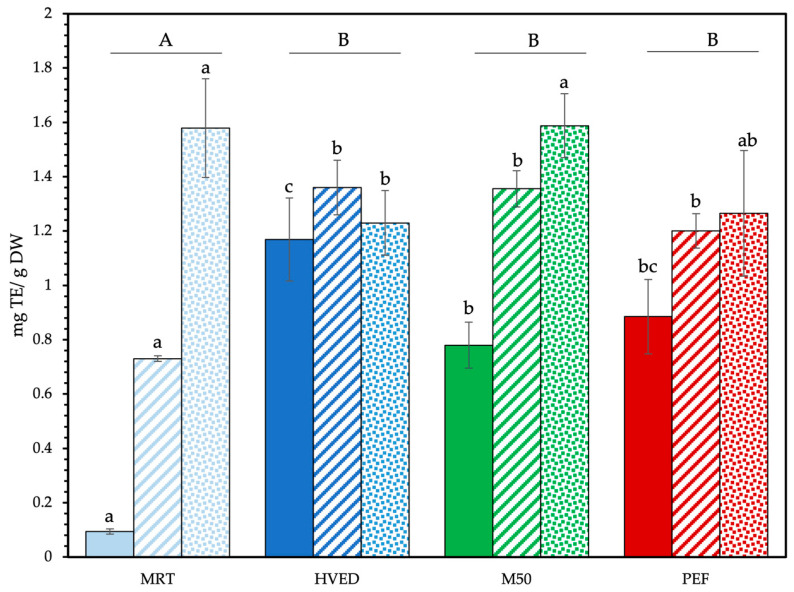
Antioxidant activity of 1:20 extracts. Solid fill: fresh GC; dashed fill: dry GC; dotted fill: powdered GC. Data are shown as mean ± SD (n = 4). Upper case letters signify differences between treatments (values averaged across state). Lower case letters denote significant differences between treatments by state. Type III ANOVA with Tukey post-hoc test.

MAAs are noted for some antioxidant capacity [65,66,67], and GC extracts have found use in cosmetics as antioxidants [12]. Moreover, HVED- and PEF-based extraction methods are compatible with the extraction of a variety of antioxidant compounds due to the low temperatures and aqueous solvents involved [68,69,70,71]. The antioxidant activity of final extracts was evaluated using the FRAP method (Figure 5). Values between 1:20 and 1:40 ratios were statistically different, with overall means higher in the 1:20 ratio.

The pretreatments increased the diffusion of antioxidant molecules from whole GC with varying efficacy according to its state. The lowest antioxidant activity (mg.Trolox Equivalents(_TE_)/g_DW_) was generally observed in fresh GC extracts and the highest in powdered GC extracts. In fresh GC, antioxidant activity was barely detectable in the control maceration at 0.09 ± 0.01 mg_TE_/g_DW_. HVED-pretreated extracts showed the highest antioxidant activity at 1.17 mg_TE_/g_DW_, a tenfold increase over the controls. This was not statistically different from PEF treatment at 0.88 ± 0.14 mg_TE_/g_DW_ (ANOVA type III, Tukey post-hoc), although superior to the other heated control (M50—0.78 ± 0.08 mg_TE_/g_DW_). PEF and M50 were not significantly different. Pretreatments also increased antioxidant activity of dry GC extracts. Control maceration resulted in antioxidant activity values of 0.73 ± 0.01 mg_TE_/g_DW_, whereas antioxidant activity was 1.36 ± 0.10 mg_TE_/g_DW_ in HVED extracts, 1.36 ± 0.07 mg_TE_/g_DW_ in heated controls, and 1.20 ± 0.06 mg_TE_/g_DW_ in PEF. The difference between pretreatments was not statistically significant. The assorted physical effects were beneficial for increased diffusion of antioxidant compounds. Powdered GC gave the highest overall antioxidant activity. Antioxidant activity was greatest in the controls, with 1.58 ± 0.18 mg_TE_/g_DW_ in MRT extracts and 1.59 ± 0.12 mg_TE_/g_DW_ in M50 extracts. This was significantly greater than that of HVED with 1.23 ± 0.12 mg_TE_/g_DW_, but not significantly different from the PEF extracts (1.27 ± 0.23 mg_TE_/g_DW_). The free radicals generated during the HVED pretreatment may have affected the antioxidant molecules in the powdered extract to a greater extent than in whole (fresh and dry) GC with extensive damage to the biomatrix.

Compared to the values of other authors, Castejon et al. [6] found FRAP of approximately 0.75 mg_TE_/g_DW_ when using UAE techniques with aqueous solvents and longer extraction times, and up to 1.15 ± 0.06 mg TE/ g _DW_ when using ethanol–water mixtures. Cavaco et al. [7] found values of 0.91 ± 0.22 ascorbic acid equivalents/g_DW_ in the FRAP assay, whereas Matos et al. did not find any activity in either FRAP or DPPH assays [46]. Although some variation may be explained by seasonality, Cavaco [7] did not find statistically significant differences with the FRAP assay, but did with an ABTS [2,2′-azinobis-(3-ethylbenzothiazoline-6-sulfonate)] assay. All antioxidant effects of the extracts were low as compared to standards, generally around 0.1% activity of equivalent weight standards.

## 4. Conclusions

The effect of HVED and PEF on MAA and protein extraction yields from fresh, dry, and powdered Gelidium algae was investigated, as was extract antioxidant activity. Efficacy of the treatment for MAA yield was dependent on the state of the algae, with the most marked effect of electrical treatments observed on fresh algae. In fresh algae, HVED MAA yields were roughly 20 times higher than in the unheated MRT control treatment and close to double the yields of the other treatments (PEF and M50). These results suggest that the intense shock waves and associated forces specific to HVED induce the best release of MAAs from fresh GC. When the algae were in dried or powdered form, electrical treatment and/or heating did not provide further benefit for MAA yields. The extract protein content followed a similar general pattern with an advantageous effect of the treatments on fresh and dry GC, with a less marked effect on powder GC. Antioxidant activity of the extracts was relatively low. FRAP antioxidant assays demonstrated that the extraction technique did not have negative effects on the antioxidant activity of the extracts when the algae were whole, and only a small effect on powdered GC. Overall, pulsed power techniques such as HVED and PEF are promising pretreatments for whole seaweeds and can be implemented directly after harvest in the initial stages of processing. Further study should focus on other molecules that were not quantified here (polyphenols, for instance) and the effect of electrical pretreatment on agar quality.

## Figures and Tables

**Table 1:** State of algae and treatment conditions.

Algal State	Solid to Liquid Ratio	Extraction Identification	Extraction Conditions
FRESH DRY POWDER	1:20 *w/v*	PEF	PEF (500 kJ/kgsol; 20 kV/cm; 620 pulses, 200 J) + maceration at room temperature
		HVED	HVED (500 kJ/kgsol; 750 J, 300 pulses) + maceration at room temperature
	1:40 *w/v*	MRT	Room temperature maceration
		M50	Maceration at 50 °C for 10 min + maceration at room temperature

**Table 2:** MAA contents in GC extracts identified and quantified using untargeted ddMS2/MS3 analysis.

		MRT	HVED	M50	PEF
∑ MAAs (mg/g_DW_)	Fresh	0.037 ± 0.002	0.81 ± 0.05	0.45 ± 0.13	0.45 ± 0.01
	Dry	0.59 ± 0.02	0.54 ± 0.05	0.57 ± 0.2	0.61 ± 0.16
	Powder	0.43 ± 0.12	0.59 ± 0.19	0.64 ± 0.05	0.61 ± 0.04
Palythine (mg/g_DW_)	Fresh	0.002 ± 0.001	0.07 ± 0.03	0.02 ± 0.02	0.03 ± 0.03
	Dry	0.04 ± 0.04	0.11 ± 0.08	0.04 ± 0.05	0.06 ± 0.07
	Powder	0.03 ± 0.02	0.05 ± 0.06	0.04 ± 0.04	0.04 ± 0.04
Shinorine (mg/g_DW_)	Fresh	0.001 ± 0.001	0.07 ± 0.05	0.04 ± 0.05	0.03 ± 0.04
	Dry	0.05 ± 0.05	0.03 ± 0.03	0.05 ± 0.06	0.06 ± 0.07
	Powder	0.04 ± 0.05	0.04 ± 0.04	0.05 ± 0.05	0.04 ± 0.05
Asterina (mg/g_DW_)	Fresh	0.034 ± 0.002	0.68 ± 0.07	0.39 ± 0.10	0.395 ± 0.002
	Dry	0.51 ± 0.04	0.37 ± 0.18	0.49 ± 0.16	0.52 ± 0.12
	Powder	0.36 ± 0.09	0.5 ± 0.17	0.56 ± 0.06	0.52 ± 0.05

**Table 3:** Relative content of MAAs in treated samples.

	MRT	HVED	M50	PEF
Fresh	2.53%	10.68%	2.73%	2.60%
Dry	4.92%	2.81%	2.40%	2.30%
Powder	2.85%	3.48%	2.61%	3.31%

**Table 4:** Protein ratio of dry extracts and percentage of extracted protein over total algal protein n = 3.

Protein Ratio of Dry Extracts				
	MRT	HVED	M50	PEF
Fresh	13.25 ± 0.18	5.97 ± 0.26	3.51 ± 0.28	3.86 ± 0.37
Dry	4.88 ± 0.13	5.14 ± 0.09	4.41 ± 0.29	4.08 ± 0.18
Powder	6.82 ± 0.10	7.36 ± 0.18	6.83 ± 0.57	6.19 ± 0.12
% Extracted Protein				
	MRT	HVED	M50	PEF
Fresh	0.77 ± 0.01	6.32 ± 0.27	4.20 ± 0.33	4.39 ± 0.42
Dry	6.57 ± 0.17	7.56 ± 0.14	6.21 ± 0.41	6.31 ± 0.28
Powder	11.16 ± 0.17	11.25 ± 0.28	10.21 ± 0.85	9.86 ± 0.20

## Data Availability

The data presented in this study are openly available in FigShare at https://doi.org/10.6084/m9.figshare.22348678.v1.

## Supplementary Materials

The following supporting information can be downloaded at: https://www.mdpi.com/article/10.3390/foods12071473/s1, Figure S1. Comparison of the absorbance of UV-absorbing compounds (MAAs) at 325 nm of the two ratios: 1:20 (top curve) and 1:40 (bottom curve). (A-C). MRT; (D-F). HVED; (G-I) M50; (J-L) PEF. Yellow areas represent the treatment period. Grey areas represent initial rehydration step. Shaded areas represent the standard deviation of subsamples (n = 2). Figure S2. Aspect of extracts fresh, dry, powdered algal extracts (from left to right) using: (A) MRT (B) HVED; (C) M50;(D) PEF. Figure S3. UV-Vis Spectra of extracts post-maceration. Insets display zoomed area between 450 and 800 nm. A/ MRT extracts B/ HVED C/ M50 D/PEF. Figure S4. Dry weight of soluble compounds extracted from GS at 1:40 ratio. Data are shown as mean ± SD (n = 3). Upper case letters signify differences between treatments (values averaged across State, n = 12). Lower case letters denote significant differences between States by Treatment n = 3). Type III ANOVA with Tukey Post-Hoc test. Figure S5. Total protein yields in different treatments. Solid fill: fresh GS. Dashed fill: dry GS. Dotted fill: powdered GS. Data are shown as mean ± SD (n = 4); Table S1. Increases of temperature during treatment (500 kJ/kg sol); Table S2. Protein content of dry extracts for both 1:20 and 1:40 ratios.
