# Supplementary material for: Extraction of Mycosporine-like Amino Acids and Proteins from the Agarophyte Gelidium corneum Using Pulsed Power Techniques
Source: Foods. 2023 Mar 30;12(7):1473. doi: 10.3390/foods12071473 (PMC10094572; doi:10.3390/foods12071473)
Supplement: Supplementary file 1 [file foods-12-01473-s001.zip › foods-2305700-supplementary.pdf]

Supplementary Materials

Table S1. Increases of temperature during treatment (500 kJ/kg sol)

State of GS	Treatment/ Ratio	Temperature increase (°C)	Maximum temperature (°C)
**Fresh**	HVED 1:20	12	30
	HVED 1:40	11	32
	PEF 1:20	34	49
	PEF 1:40	35	54
**Dry**	HVED 1:20	16	34
	HVED 1:40	16	35
	PEF 1:20	43	63
	PEF 1:40	41	56
**Powder**	HVED 1:20	15	33
	HVED 1:40	15	35
	PEF 1:20	36	56
	PEF 1:40	36	58

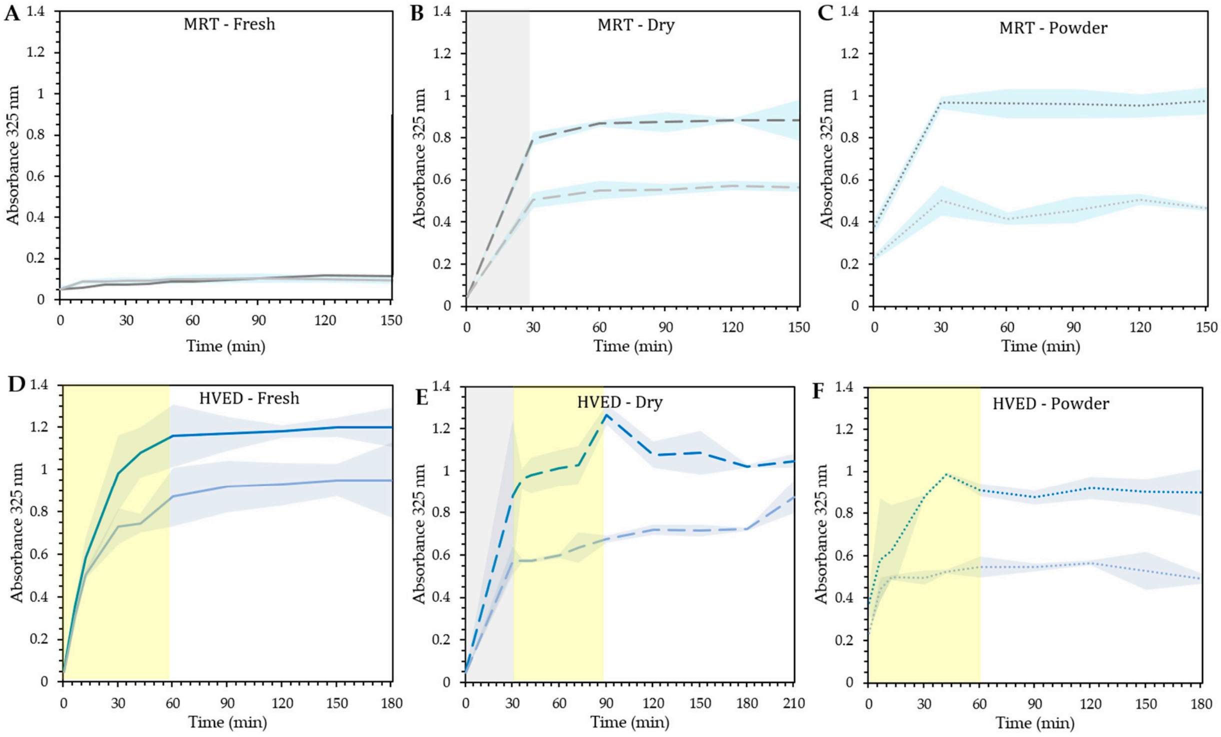

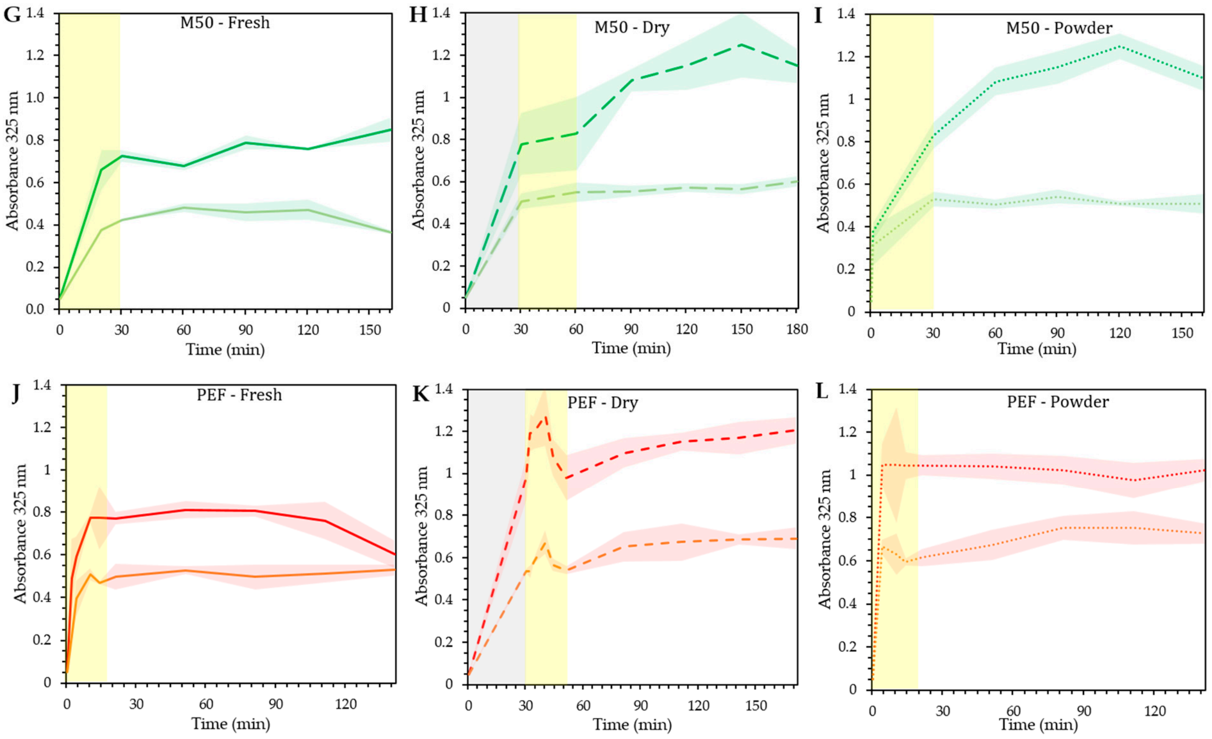

Figure S1. Comparison of the absorbance of UV-absorbing compounds (MAAs) at 325 nm of the two ratios: 1:20 (top curve) and 1:40 (bottom curve). (A-C). MRT; (D-F). HVED; (G-I) M50; (J-L) PEF. Yellow areas represent the treatment period. Grey areas represent initial rehydration step. Shaded areas represent the standard deviation of subsamples (n=2).

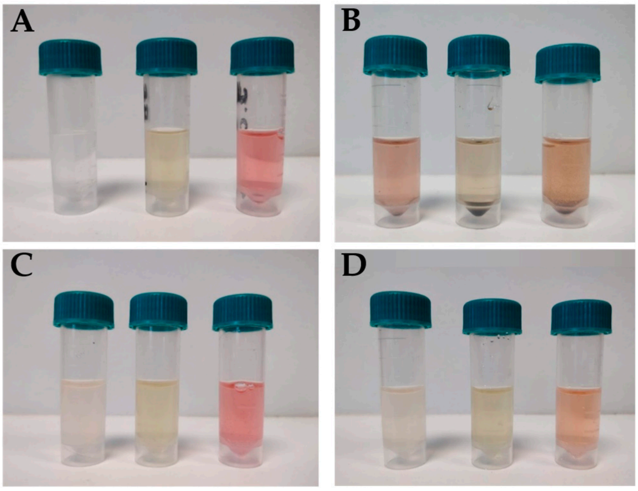

Figure S2. Aspect of extracts fresh, dry, powdered algal extracts (from left to right) using: (A) MRT (B) HVED; (C) M50;(D) PEF.

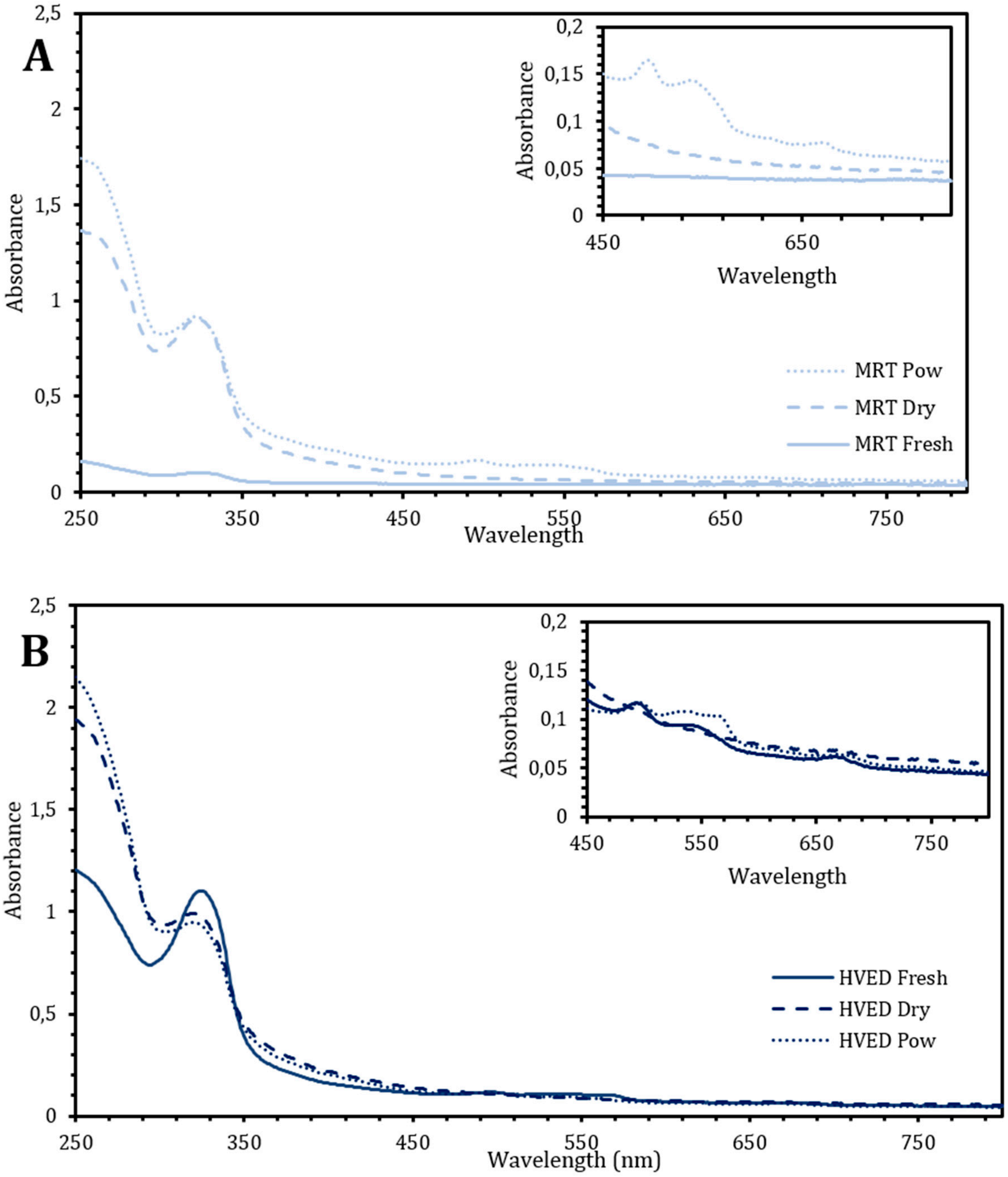

A
Absorbance
Wavelength
MRT Pow
MRT Dry
MRT Fresh
B
Absorbance
Wavelength (nm)
Wavelength
HVED Fresh
HVED Dry
HVED Pow

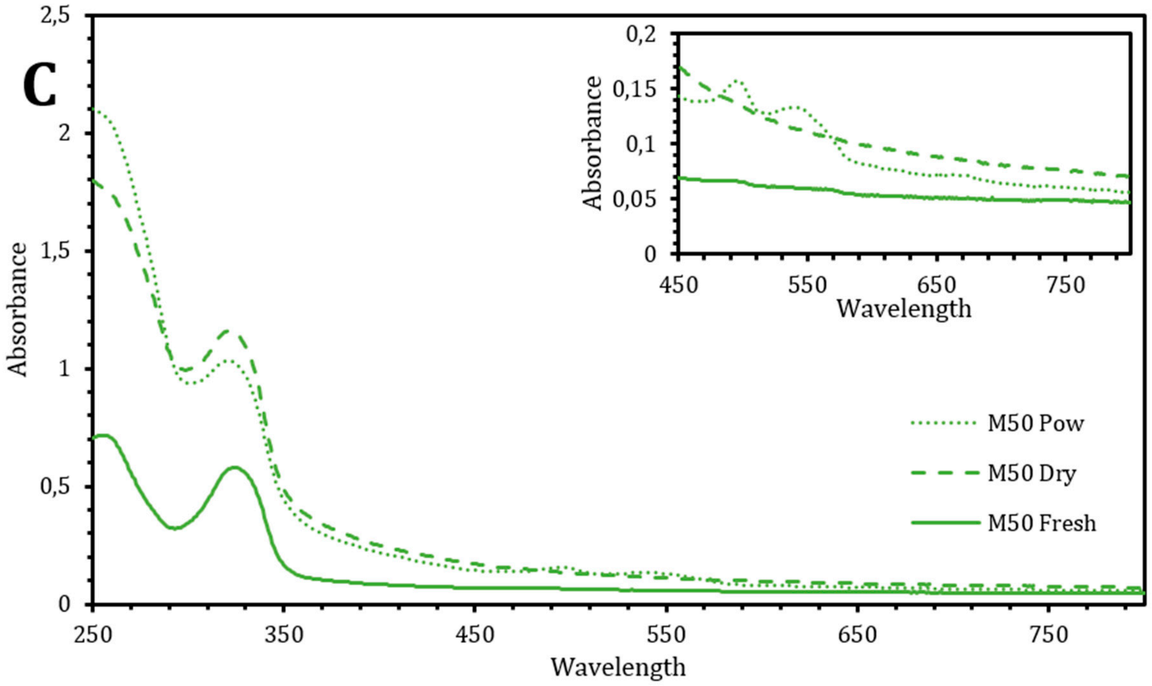

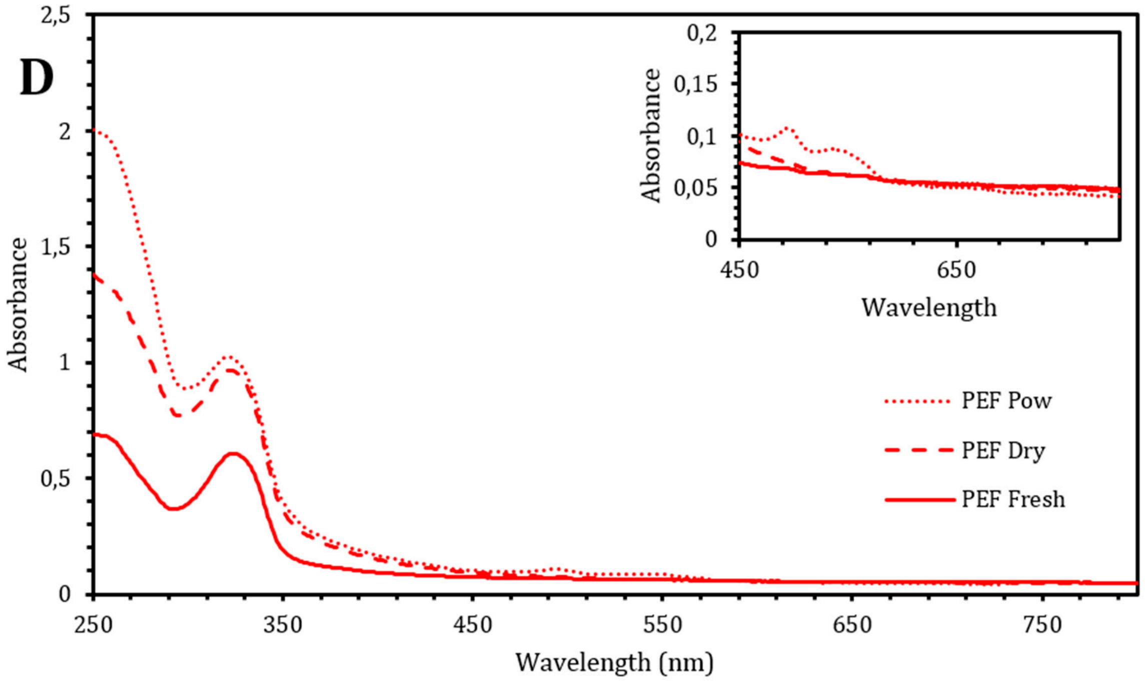

Figure S3. UV-Vis Spectra of extracts post-maceration. Insets display zoomed area between 450 and 800 nm. A/ MRT extracts B/ HVED C/ M50 D/PEF

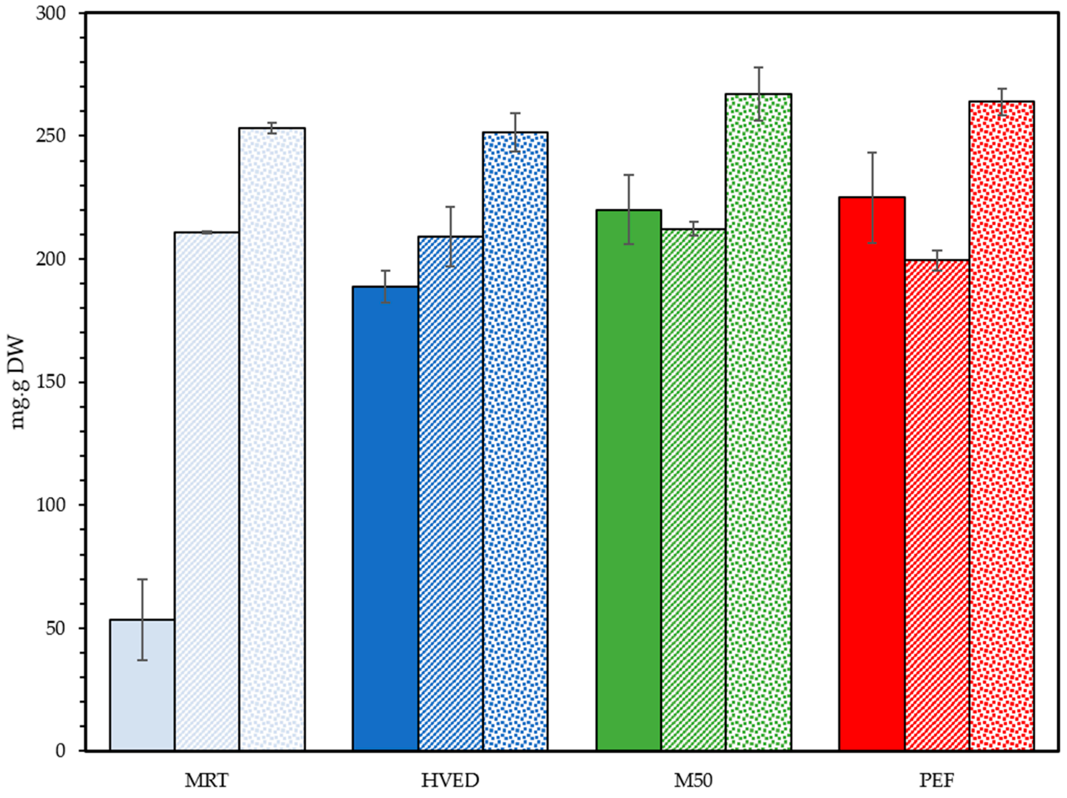

Figure S4. Dry weight of soluble compounds extracted from GS at 1:40 ratio. Data are shown as mean ± SD (n=3). Upper case letters signify differences between treatments (values averaged across State, n=12). Lower case letters denote significant differences between States by Treatment n=3). Type III ANOVA with Turkey Post-Hoc test.

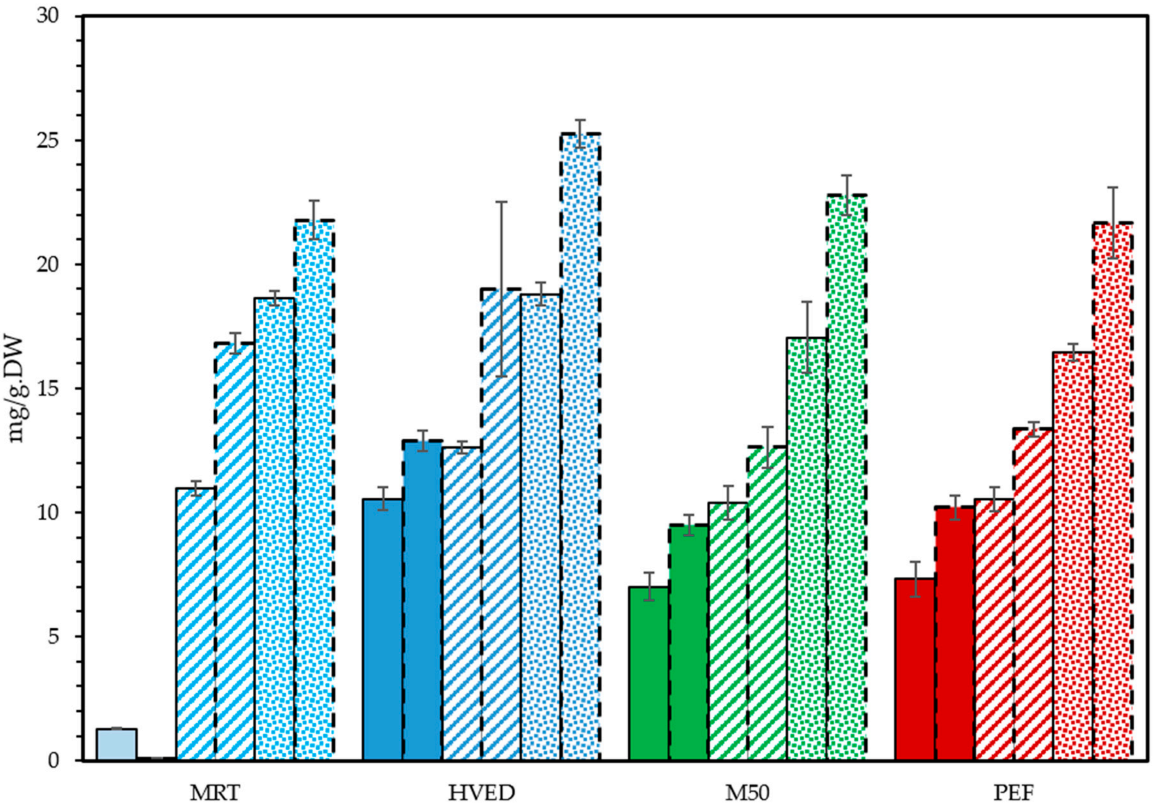

Figure S5. Total protein yields in different treatments. Solid fill: fresh GS. Dashed fill: dry GS. Dotted fill: powdered GS. Data are shown as mean ± SD (n=4)

Table S2. Protein content of dry extracts for both 1:20 and 1:40 ratios.

		Protein Content of Dry Extracts				
		**MRT**	**HVED**	**M50**	**PEF**	**Average**
Fresh	1:20	13.3 ± 0.2%	8.8 ± 0.3%	3.5 ± 0.3%	3.9 ± 0.4%	6.2 ± 3.4%
	1:40	4.8 ± 1.3%	6.8 ± 0.2%	4.3 ± 0.2%	4.2 ± 0.2%	
Dry	1:20	6.0 ± 0.3%	6.5 ± 0.3%	5.4 ± 0.2%	5.2 ± 0.2%	7.0±1.0%
	1:40	8.0 ± 0.2%	9.1 ± 1.7%	8.8 ± 0.4%	8.2 ± 0.1%	
Powder	1:20	6.8 ± 0.1%	7.4 ± 0.2%	6.8 ± 0.6%	6.2 ± 0.1%	7.7±1.3%
	1:40	8.6 ± 0.3%	10.0 ± 0.2%	8.5 ± 0.3%	7.0 ± 0.5%	
Average		8.0 ± 0.4%	8.1 ± 0.5%	6.0 ± 0.3	5.7 ± 0.3	
